# Tuberculosis Progression Does Not Necessarily Equate with a Failure of Immune Control

**DOI:** 10.3390/microorganisms7070185

**Published:** 2019-06-27

**Authors:** David G. Russell

**Affiliations:** Microbiology and Immunology, College of Veterinary Medicine, Cornell University, Ithaca, NY 14853, USA; Dgr8@cornell.edu; Tel.: +1-607-253-4272

**Keywords:** mycobacterium, tuberculosis, *Mycobacterium tuberculosis*, macrophage

## Abstract

Despite the obvious impact of tuberculosis on global health, there is currently no effective vaccine and there is increasing resistance against established front-line drug regiments. Our current understanding of disease progression in tuberculosis is shaped by data collected from the failure of immune control. We feel that this represents a biased approach, which constrains our capacity to understand both disease control and progression. In this opinion piece, we re-examine these questions in the context of recently published data from fluorescent bacterial reporter strains and the analysis of the different macrophage lineages present at sites of infection. We believe that this analysis provides alternative models for disease progression, which are not addressed through current vaccine or immune-therapeutic strategies.

## 1. Introduction.

Tuberculosis is an ancient disease caused by *Mycobacterium tuberculosis* (Mtb) that has co-evolved with its human host since before man emerged from Africa [1,2] Ancestral strains are thought to be represented by several of the current Mtb complex strains still found in Central and West Africa, such as *M. africanum*. For a large part of our evolution, we have existed as small hunter/gatherer groups and it is surmised that Mtb, as an inducer of a chronic sustained infection, would have evolved to be capable of infecting many members of a group yet only generating disease in a limited set of individuals at any given time. In this way, a human-specific pathogen lacking an animal reservoir could expand and be maintained in small population units in a relatively balanced state. However, this is not a progression to symbiosis. In order to transmit and complete its life cycle, Mtb has to make its host sick [3]. Active tuberculosis is the completion of the pathogen’s life cycle. It is extremely efficient and in most individuals in the absence of treatment, it is ultimately fatal. Mtb, like all organisms on the planet, is driven by the “selfish gene” principle and if damaging its host comes with increased fecundity, that is the direction in which it will be selected. 

## 2. To Progress or Not to Progress

Thus, what determines the transition from a chronic to an active state of infection? It is estimated that approximately 23% of the world’s population is infected with Mtb, but the majority harbor the pathogen in a non-active disease state, which is known as latent tuberculosis infections (LTBI) [4]. There is debate about the average duration of latency and the relative frequency of reactivation of latent disease versus re-infection of individuals [5]. In areas of high transmission density, it would appear that the latter is much more common than we have appreciated previously [6]. 

The ability to assess the difference between reactivation versus re-infection is critical for biomarker studies that seek to identify immune correlates with the capacity to predict disease progression within a population. Unfortunately, most of these studies have been conducted in South Africa because of its high disease burden and patient accessibility and its developed clinical research capacity. However, it is challenging to conduct such studies in this population because the high transmission pressure in many South African communities will lead to re-infection that will misinform attempts to identify predictive correlates of immune status. 

However, what these studies do have is the capacity to generate increasingly sensitive diagnostic indicators of the early events associated with disease progression. Early peripheral transcriptomics analysis of peripheral blood identified a neutrophil signature that was associated with progression to active disease [7]. Since these initial studies, the analyses have become increasingly more sophisticated and sensitive. However, I feel that they are reliant on the detection of a disease process that has already been initiated [8,9,10]. For that reason, I believe that they are diagnostic biomarkers but are not predictive. Undoubtedly, such readouts are of great value in the early identification of individuals on the pathway to the development of clinical disease and will help to direct the initiation of early treatment. However, these readouts are unlikely to be of value in the assessment of immune status in the absence of disease progression. Therefore, we are still working blindly when it comes to correlates of immune protection with the capacity to inform vaccine development strategies. 

## 3. What Do We Know of Immune Protection? 

The majority of our knowledge regarding immune protection has come from the study of immune failure [11]. We know how different knockout mouse strains behave after being challenged with Mtb and we have a list of human genes that correlate with differing degrees of susceptibility to active tuberculosis. However, the extrapolation of data from immune failure to the identification of desirable characteristics of protection and the use of these correlates for the development of vaccine programs is optimistic at best. 

If you take a wheel off a car, it does not work very well. However, there is no guarantee that adding a fifth wheel to the car is going to improve its performance. Unfortunately, this appears to be the rationale behind many of the vaccine strategies that use peripheral production of IFN-γ as a surrogate for vaccine efficacy. Clearly, IFN-γ is required but it is not sufficient in itself. In general, the vaccine community has tended to focus almost exclusively on the functionality of different lymphocyte subsets and how the loss of a certain T-cell subsets and/or cytokines impacts negatively on acquired immune protection. 

Such approaches are pretty much restricted to the generation of correlative data that documents failure of immune control rather than the identification of pathways of improved control. 

## 4. Immune Control Is Filtered Through the Host Phagocyte

Although vaccines modify the acquired immune response, the impact of the acquired immune response must ultimately be translated through the response of the infected host macrophages. The world of macrophage biology has undergone somewhat of a re-birth over the past five years. We used to believe that all macrophages were hemopoetically-derived and started with a neutral M0 phenotype, which could be driven to either classically-activated (M1) or alternatively-activated (M2) states through exposure to type 1 (IFN-γ) or type 2 (IL-4, IL-13) cytokines. Recent cell fate mapping studies in mice has shown that this is not to be the case [12,13]. Most notably, for tuberculosis, alveolar macrophages are fetal stem cell-derived cells that populate the tissue early in development and represent a long-lived, self-replenishing tissue resident population lineage. Indeed, in humans 3–4 years post-lung transplant, 85–90% of the alveolar macrophages are donor-derived [14,15]. 

The significance of the different myeloid populations in tuberculosis was demonstrated recently through the use of fluorescent fitness reporter strains of Mtb in experimental murine infections [16]. Following a short-term Mtb infection (14 days), the bacilli were found to be roughly equally distributed between the resident alveolar macrophages and recruited interstitial macrophage populations. Using the fluorescent fitness readouts [17,18], we found that the bacteria in the alveolar macrophages exhibited higher indicators of replication and lower indicators of stress than those bacteria in the recruited interstitial macrophages. We selectively depleted both macrophage subsets individually using clodronate liposomes delivered into the lung airways or peripheral blood. Depletion of the alveolar macrophages resulted in a log reduction in bacterial burden, while depletion of the peripheral blood monocytes that give rise to the interstitial macrophages resulted in a log increase in bacterial burden [16]. As Mtb only infects 1–2% of the total macrophage population in the lung, we conclude that the bacterial burden is directly influenced by the identity of its host macrophage population.

Transcriptional and metabolic profiling of the two infected phagocyte populations demonstrated that the interstitial macrophages were committed to glycolysis, whereas the alveolar macrophages demonstrated a marked bias towards fatty acid oxidation and mitochondrial respiration. Intriguingly, intoxication of interstitial macrophages with the non-hydrolysable glucose analog 2-deoxyglucose, both in vitro and in vivo, led to an enhancement of Mtb growth, whereas treatment of macrophages with the fatty acid oxidation inhibitor Etomoxir reduced bacterial growth in vitro. 

These data demonstrate the tight metabolic relationship that exists between Mtb and its host phagocytes [16,19]. However, more significantly, they provide an alternative model of disease progression that does not come from failure of the acquired immune response. Expansion of the permissive alveolar macrophage population would support increased bacterial growth. 

## 5. Is Host Macrophage Ontogeny a Missing Link in Tuberculosis Progression? 

**Figure 1:**
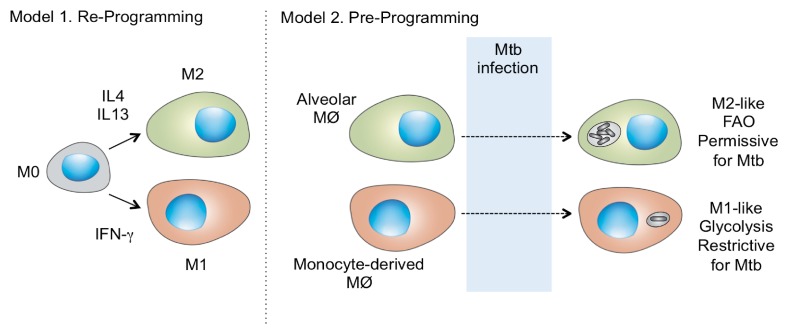
**Diagrammatic representation of an alternate model for macrophage polarization.** Model 1 illustrates the current paradigm whereby neutral, or M0, macrophages can be programmed by cytokine exposure to adopt either an M1, classically-activated, or an M2, alternatively-activated, state. In contrast, in Model 2, the macrophage lineages are pre-programmed to respond divergently to the same cytokine milieu. As we have demonstrated previously, the immune environment of the murine TB granuloma results in the resident alveolar macrophages adopting an M2-like phenotype, while the recruited interstitial macrophages adopt a classically-activated M1-like phenotype [16]. The alveolar macrophages are more permissive to bacterial growth and the expansion of this host cell population could result in bacterial growth and disease progression. Modified from [16].

Tuberculosis is not the only disease in which macrophage ontogeny appears to play a significant role in disease progression or maintenance. In cutaneous leishmaniasis, the resident dermal macrophages support parasite expansion in comparison to the recruited, blood monocyte-derived macrophages [20]. Significantly, in both infections, the permissive tissue resident macrophage populations were driven into replication by the infection. This suggests that an increase in pathogen burden could be driven by the selective expansion of permissive, tissue resident macrophage lineages as represented in Figure 1. Furthermore, such a phenomenon could occur in the face of a highly-effective controlling immune response. 

By limiting our models of tuberculosis disease progression to the failure of immune control we are restricting our capacity to detect alternative explanations, such as the gain of permissiveness (namely, the expansion of host macrophages that are non-responsive or respond divergently to type 1 cytokines). Given the failure of our vaccine strategies based on Th1 immunity, we believe that it is vital to explore alternative routes to disease progression. We would argue that the use of fluorescent Mtb fitness reporters [16,17,18] circumvents the need to rely on indirect immune correlates through the provision of direct readouts of the fitness and the replication status of the pathogen itself. 

## 6. Final Comments

These data bring into focus two other issues that need to be reassessed. It has long been known that disease progression is determined at the level of the individual granuloma and not systemically. This has always been perceived as a limitation to the search for peripheral biomarkers of disease. However, this challenge is now even greater when one acknowledges that the cell lineages that appear responsible for bacterial expansion are tissue-specific lineages that lack a peripheral counterpart. Secondly, we have always thought that cytokines would elicit comparable responses in different macrophage populations, but this appears not to be the case. Despite the fact that both alveolar and interstitial macrophage lineages experience the same immune milieu, they clearly respond divergently [16,21] as shown in Figure 1. Is this a product of epigenetic control? Whatever the mechanistic explanation, the divergent responses of macrophage lineages to the same immunological environment represents an additional challenge to vaccine-mediated control. 

If we continue to rely on immune correlates of “protection” to support ongoing vaccine design, I believe that we are destined to repeat our recent failures. As a field, we need less biased readouts of disease progression and microbial readouts are always going to be more directly informative and reliable than immune correlates. 

## References

[1] Hershberg R., Lipatov M., Small P.M., Sheffer H., Niemann S., Homolka S., Roach J.C., Kremer K., Petrov D.A., Feldman M.W. (2008). High functional diversity in *Mycobacterium tuberculosis* driven by genetic drift and human demography. PLoS Biol..

[2] Wirth T., Hildebrand F., Allix-Beguec C., Wolbeling F., Kubica T., Kremer K., van Soolingen D., Rusch-Gerdes S., Locht C., Brisse S. (2008). Origin, spread and demography of the *Mycobacterium tuberculosis* complex. PLoS Pathog..

[3] Russell D.G., Barry C.E., Flynn J.L. (2010). Tuberculosis: what we don’t know can and does, hurt us. Science.

[4] Houben R.M., Dodd P.J. (2016). The Global Burden of Latent Tuberculosis Infection: A Re-estimation Using Mathematical Modelling. PLoS Med..

[5] Behr M.A., PEdelstein H., Ramakrishnan L. (2018). Revisiting the timetable of tuberculosis. BMJ.

[6] Houben R.M., Crampin A.C., Ndhlovu R., Sonnenberg P., Godfrey-Faussett P., Haas W.H., Engelmann G., Lombard C.J., Wilkinson D., Bruchfeld J. (2011). Human immunodeficiency virus associated tuberculosis more often due to recent infection than reactivation of latent infection. Int. J. Tuberc. Lung Dis..

[7] Berry M.P., Graham C.M., McNab F.W., Xu Z., Bloch S.A., Oni T., Wilkinson K.A., Banchereau R., Skinner J., Wilkinson R.J. (2010). An interferon-inducible neutrophil-driven blood transcriptional signature in human tuberculosis. Nature.

[8] Fiore-Gartland A., Carpp L.N., Naidoo K., Thompson E., Zak D.E., Self S., Churchyard G., Walzl G., Penn-Nicholson A., Scriba T.J. (2018). Considerations for biomarker-targeted intervention strategies for tuberculosis disease prevention. Tuberculosis.

[9] Musvosvi M., Duffy D., Filander E., Africa H., Mabwe S., Jaxa L., Bilek N., Llibre A., Rouilly V., Hatherill M. (2018). T-cell biomarkers for diagnosis of tuberculosis: candidate evaluation by a simple whole blood assay for clinical translation. Eur. Respir. J..

[10] Suliman S., Thompson E., Sutherland J., Weiner Rd J., Ota M.O.C., Shankar S., Penn-Nicholson A., Thiel B., Erasmus M., Maertzdorf J. (2018). GC6-74 and ACS Cohort Study groups. Four-gene Pan-African Blood Signature Predicts Progression to Tuberculosis. Am. J. Respir. Crit. Care Med..

[11] North R.J., Jung Y.J. (2004). Immunity to tuberculosis. Annu. Rev. Immunol..

[12] Ginhoux F., Guilliams M. (2016). Tissue-Resident Macrophage Ontogeny and Homeostasis. Immunity.

[13] Ginhoux F., Jung S. (2014). Monocytes and macrophages: developmental pathways and tissue homeostasis. Nat. Rev. Immunol..

[14] Eguiluz-Gracia I., Schultz H.H., Sikkeland L.I., Danilova E., Holm A.M., Pronk C.J., Agace W.W., Iversen M., Andersen C., Jahnsen F.L. (2016). Long-term persistence of human donor alveolar macrophages in lung transplant recipients. Thorax.

[15] Nayak D.K., Zhou F., Xu M., Huang J., Tsuji M., Hachem R., Mohanakumar T. (2016). Long-Term Persistence of Donor Alveolar Macrophages in Human Lung Transplant Recipients That Influences Donor-Specific Immune Responses. Am. J. Transpl..

[16] Huang L., Nazarova E.V., Tan S., Liu Y., Russell D.G. (2018). Growth of *Mycobacterium tuberculosis* in vivo segregates with host macrophage metabolism and ontogeny. J. Exp. Med..

[17] Sukumar N., Tan S., Aldridge B.B., Russell D.G. (2014). Exploitation of *Mycobacterium tuberculosis* reporter strains to probe the impact of vaccination at sites of infection. PLoS Pathog..

[18] Tan S., Sukumar N., Abramovitch R.B., Parish T., Russell D.G. (2013). *Mycobacterium tuberculosis* responds to chloride and pH as synergistic cues to the immune status of its host cell. PLoS Pathog.

[19] Russell D.G., Huang L., VanderVen B.C. (2019). Immunometabolism at the interface between macrophages and pathogens. Nat. Rev. Immunol..

[20] Lee S.H., Charmoy M., Romano A., Paun A., Chaves M.M., Cope F.O., Ralph D.A., Sacks D.L. (2018). Mannose receptor high, M2 dermal macrophages mediate nonhealing Leishmania major infection in a Th1 immune environment. J. Exp. Med..

[21] Mould K.J., Barthel L., Mohning M.P., Thomas S.M., McCubbrey A.L., Danhorn T., Leach S.M., Fingerlin T.E., O’Connor B.P., Reisz J.A. (2017). Cell Origin Dictates Programming of Resident versus Recruited Macrophages during Acute Lung Injury. Am. J. Respir. Cell. Mol. Biol..

